# Incremental prognostic value of a preoperative immune-metabolic-inflammatory score for disease-free survival in endometrial cancer: A retrospective cohort study

**DOI:** 10.1097/MD.0000000000050354

**Published:** 2026-08-21

**Authors:** Shuhuan Li, Juan Li, Yuer Sun, Jiayu Chen, Jing Yan, Miaomiao Nie, Mei Wu, Yawen Shao, Zhenzhen Wu

**Affiliations:** a First School of Clinical Medicine, Gansu University of Chinese Medicine, Lanzhou, Gansu, China; b Gansu Provincial Maternal and Child Health Hospital (Gansu Provincial Central Hospital), Lanzhou, Gansu, China.

**Keywords:** disease-free survival, endometrial cancer, immune-metabolic-inflammatory score, internal validation, prognostic model, systemic inflammation

## Abstract

The systemic immune-inflammation index, fibrinogen, triglyceride/high-density lipoprotein cholesterol ratio, and prognostic nutritional index have each been linked to endometrial cancer outcomes, but their optimal combination is uncertain. We compared alternative formulations and evaluated incremental prognostic value for disease-free survival (DFS). This single-center cohort included 853 surgically treated patients. Four biomarkers and 3 risk-aligned, equally weighted scores were compared: the 4-component immune-metabolic-inflammatory score (IMIS), 3-component simplified IMIS (sIMIS), and a 2-component score. Evaluation included Cox association strength, discrimination, prediction error, out-of-fold (OOF) calibration, and exploratory decision curves. The selected score was assessed alone and after addition to a 4-variable pathology reference model using 1000 patient-level bootstrap samples and stratified 10-fold OOF prediction. Alternative formulations were retained as sensitivity analyses. Median follow-up was 4.93 years, with 94 DFS events. All 4 biomarkers were associated with DFS. sIMIS had the largest likelihood-ratio statistic and numerically highest optimism-corrected and OOF C-indices, while the alternative formulations yielded broadly concordant performance. Each 1 – standard deviation increase in sIMIS was associated with a higher hazard of a DFS event (multivariable hazard ratio, 1.65; 95% confidence interval [CI], 1.45–1.88; *P* < .001). The pathology + sIMIS model had optimism-corrected and OOF C-indices of 0.820 and 0.819. Relative to pathology alone, the paired C-index difference was 0.0664 (95% CI, 0.0330–0.1025). The 5-year Brier-score gain was 0.0104 (95% CI, 0.0044–0.0165); the 3-year CI included zero. Treatment-by-risk interaction estimates were imprecise and were not statistically significant. In this single-center development cohort, sIMIS provided the most favorable overall balance of prognostic performance, component count, and construct coverage under internal resampling, although the alternative formulations performed broadly similarly. sIMIS remains a candidate risk marker rather than an established clinical or treatment-selection tool; external validation and comparison with contemporary molecularly integrated risk models are required.

## 1. Introduction

Endometrial cancer is one of the most common gynecological malignancies worldwide, and its incidence continues to increase.^[1]^ Although most patients are diagnosed with early-stage disease, recurrence risk remains heterogeneous, particularly among those with advanced-stage, high-grade tumors, non-endometrioid histology, abnormal p53 status, or other adverse molecular features. Contemporary prognostic assessment therefore integrates anatomical, pathological, and increasingly molecular information.^[2–5]^ Nevertheless, patients with apparently similar tumor characteristics may experience different outcomes, suggesting that routinely measurable host-related factors could provide complementary prognostic information.

Several preoperative blood-based markers have been associated with endometrial cancer characteristics or outcomes. Systemic immune-inflammation index (SII) combines platelet, neutrophil, and lymphocyte counts and reflects the balance between systemic inflammation and antitumor immunity.^[6,7]^ Fibrinogen (FIB) is an acute-phase coagulation protein that may capture interactions among inflammation, hemostasis, and tumor progression, and elevated preoperative levels have been associated with adverse outcomes in endometrial cancer.^[8]^ Prognostic nutritional index (PNI) integrates serum albumin and lymphocyte count and summarizes nutritional and immune status; lower PNI has been associated with poorer overall and endometrial cancer-specific survival.^[4]^ Related hemoglobin, albumin, lymphocyte, and platelet score and Controlling Nutritional Status score indices have also shown prognostic associations in endometrial cancer.^[9,10]^ Triglyceride/high-density lipoprotein cholesterol (TG/HDL-C) is a marker of dyslipidemia that has been associated with endometrial cancer presence and clinicopathological severity,^[11]^ and limited unadjusted survival stratification has been reported within a composite biomarker study^[12]^; its adjusted association with disease-free survival (DFS) remains underexplored. Broader metabolic syndrome scores have also been associated with nodal metastasis.^[13]^ These markers represent related but nonidentical host domains and are available before surgery without specialized molecular testing. A composite score may reduce dependence on any single noisy measurement and summarize complementary biological information.^[12,14,15]^ However, adding more components does not necessarily improve prediction. A useful composite should retain prognostic information while minimizing redundancy, avoiding unstable outcome-derived weights, and remaining interpretable and reproducible. We, therefore, specified a full 4-component immune-metabolic-inflammatory score (IMIS) integrating SII, FIB, TG/HDL-C, and PNI and compared it with reduced formulations rather than assuming that the most inclusive score would be preferable.

The study had 3 sequential objectives. First, we evaluated the 4 literature-informed biomarkers and 3 candidate score formulations to identify a parsimonious primary score for subsequent modeling. Second, after candidate evaluation, we quantified the association between the selected score and DFS and assessed its incremental prognostic performance beyond a 4-variable pathology reference model under patient-level bootstrap and 10-fold out-of-fold (OOF) internal validation; the other formulations were retained as sensitivity analyses. Third, after completing the prognostic analyses, we explored whether the association between recorded postoperative adjuvant therapy and DFS differed across OOF combined-model risk strata. The treatment analyses were explicitly considered hypothesis-generating and not causal.

## 2. Materials and methods

### 2.1. Study design and population

This single-center retrospective cohort included consecutive patients with pathologically confirmed endometrial cancer treated at Gansu Provincial Maternal and Child Health Hospital (Gansu Provincial Central Hospital) between March 2016 and March 2022. All patients underwent primary surgical treatment with staging procedures, defined as total hysterectomy with bilateral salpingo-oophorectomy, with or without lymph node assessment, including sentinel lymph node biopsy, pelvic lymph node dissection, and/or para-aortic lymphadenectomy when performed. The exclusion criteria were as follows: preoperative acute infection (n = 4); history of other malignancies (n = 15); concomitant immune or hematological disorders (n = 5); incomplete follow-up data (n = 41); and missing clinical information (n = 123). Ultimately, 853 patients with endometrial cancer were included. The institutional ethics committee of Gansu Provincial Maternal and Child Health Hospital (Gansu Provincial Central Hospital) approved the study (approval no. [2022] GSFY Ethics [48]) and waived the requirement for informed consent because the data were retrospectively collected and anonymized.

### 2.2. Data collection and outcome

Demographic characteristics, comorbidities, preoperative laboratory measurements, surgical pathology, p53 status, postoperative treatment, and follow-up information were extracted from the medical record. Laboratory measurements used to calculate candidate biomarkers were obtained within 10 days before surgery. Histological type was consistently classified as endometrioid or non-endometrioid. Complete molecular classification was unavailable: polymerase epsilon exonuclease domain-mutated subtype mutation and mismatch-repair status were not recorded, precluding assignment of all contemporary molecular subgroups, including no specific molecular profile subtype. Lymphovascular space invasion (LVSI) was available as a binary variable (absent or present). The primary outcome was DFS, measured from the date of surgery to the 1st documented recurrence or progression, or death from any cause. Patients without an event were censored at the date of last follow-up. The final analytic dataset contained no missing values for the score components, primary pathological predictors, event status, or follow-up time.

### 2.3. Candidate biomarkers and construction of candidate scores

Four biomarkers were selected on the basis of previously reported associations with endometrial cancer outcomes and their representation of complementary host domains: SII, FIB, TG/HDL-C, and PNI. SII was calculated as platelet count (×10^9^/L) multiplied by neutrophil count (×10^9^/L) and divided by lymphocyte count (×10^9^/L). TG/HDL-C was calculated using triglyceride and HDL-C concentrations in mmol/L. PNI was calculated as serum albumin (g/L) plus 5 times the lymphocyte count (×10^9^/L), and FIB was recorded in g/L. Each component was standardized as *z*(*x*) = (*x* − mean)/SD.^[16]^ Higher SII, FIB, and TG/HDL-C and lower PNI were aligned to the direction of higher risk. The full 4-component IMIS was defined as IMIS = *z*(SII) + *z*(FIB) + *z*(TG/HDL-C) − *z*(PNI). Two reduced formulations were also evaluated: simplified immune-metabolic-inflammatory score (sIMIS) = *z*(SII) + *z*(TG/HDL-C) − *z*(PNI), and the 2-component score = *z*(TG/HDL-C) − *z*(PNI). Risk-aligned equal weights were used to preserve transparency and avoid estimating outcome-derived component coefficients in a cohort with 94 events. Full-cohort standardization parameters and final formulas are reported in Table S1, Supplemental Digital Content 1. For effect estimation, each composite score was rescaled to its own SD so that hazard ratios (HRs) represented a 1 − SD increase.

### 2.4. Candidate-marker and score-formulation evaluation

The 4 individual biomarkers and 3 candidate score formulations were evaluated before the downstream primary prognostic analyses. Comparisons included the HR per 1 − SD risk-aligned increase, likelihood-ratio chi-square statistic from the score-only Cox model, Harrell C-index, inverse probability of censoring weighting (IPCW) area under the time-dependent receiver operating characteristic curve (AUC) at 3 and 5 years, IPCW Brier score at the same horizons, OOF calibration, and exploratory OOF decision curves. Candidate-score selection was based on the overall pattern of association strength, internally assessed prognostic performance, component count, and coverage of relevant biological constructs rather than on a single metric or *P* value. The formulation carried forward under this framework was designated the primary score, and the other 2 formulations were retained as sensitivity analyses.

### 2.5. Association and primary prognostic models

**Figure 1.**
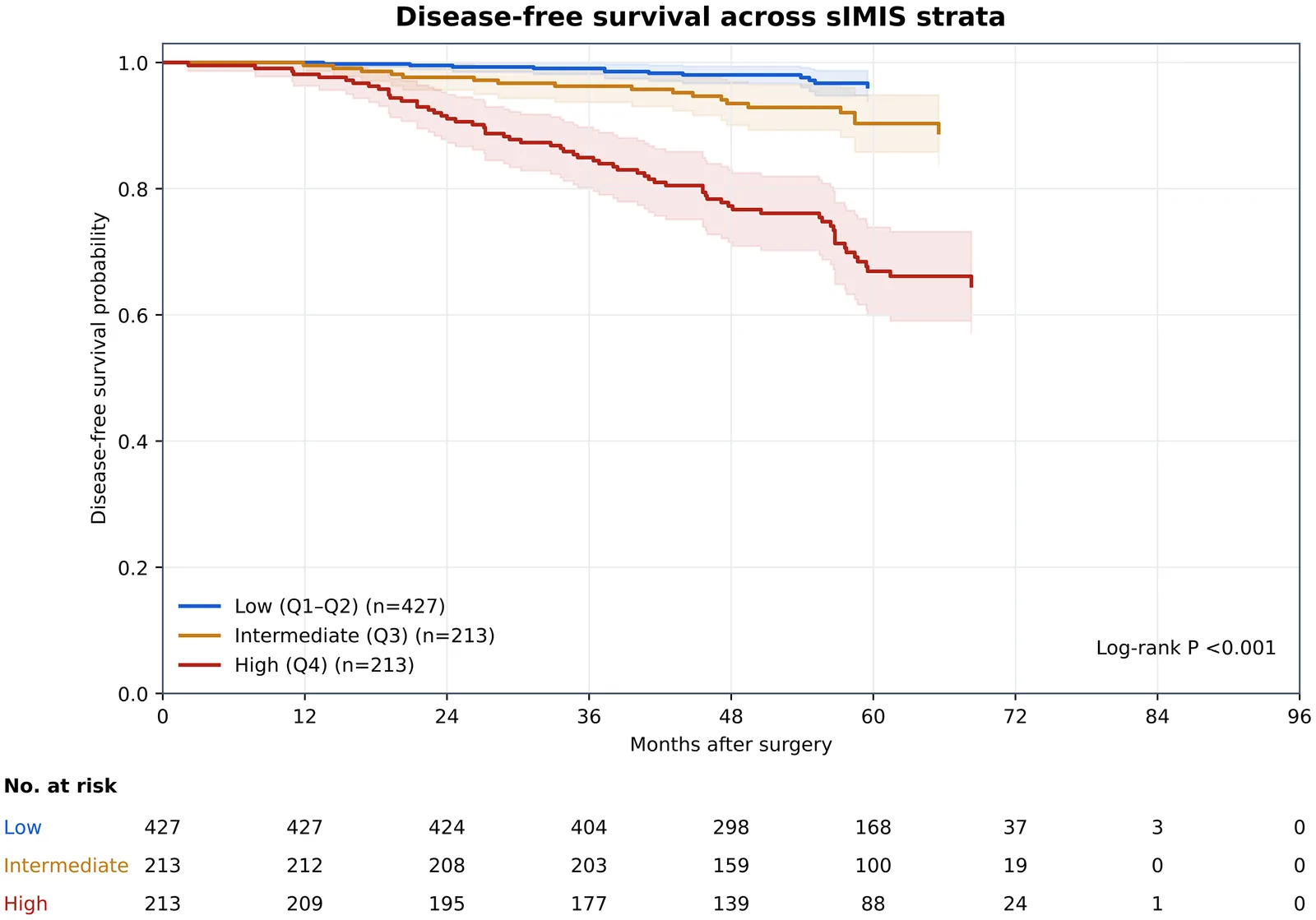
Disease-free survival across sIMIS strata. Kaplan–Meier estimates of DFS for the low (*Q*1–*Q*2; n = 427), intermediate (*Q*3; n = 213), and high (*Q*4; n = 213) sIMIS strata, with 95% confidence bands and numbers at risk. These quartile-based groups are distinct from the OOF combined-model risk strata used in the treatment analyses. DFS = disease-free survival, OOF = out-of-fold, sIMIS = simplified immune-metabolic-inflammatory score.

Continuous variables are summarized as mean ± SD or median (interquartile range [IQR]), and categorical variables as counts and percentages. Median follow-up was estimated using the reverse Kaplan–Meier method. Univariable and multivariable Cox proportional hazards models were used to estimate HRs and 95% confidence intervals (CIs). Age was modeled continuously, with the HR expressed per 1-year increase. Variables with a univariable *P* value <.05 were entered into the multivariable association model. Following candidate evaluation, the score with the most favorable overall predictive performance was selected for subsequent analyses. Three primary prognostic models were then fitted: the selected score alone, the pathology reference model, and the pathology model incorporating the selected score. Discrimination was assessed with the Harrell C-index and cumulative/dynamic IPCW AUC at 36 and 60 months. Prediction error was assessed with IPCW Brier scores at the same horizons. For descriptive survival analysis, the best-performing score quartiles were collapsed into low (*Q*1–*Q*2), intermediate (*Q*3), and high (*Q*4) strata and compared using a log-rank test.^[17]^ This quartile-based grouping of the best-performing score was used only for Figure 1 and was distinct from the OOF combined-model risk strata used in the treatment analyses. A nomogram was constructed from the fitted pathology plus best-performing score Cox model as an illustrative representation of the candidate model. The proportional hazards assumption was evaluated using scaled Schoenfeld residuals with the rank time transform. Variable-level tests were reported for the univariable models, the multivariable association model, and the primary and sensitivity prognostic models. Global rows were treated as approximate sums of variable-level statistics.

### 2.6. Internal validation, calibration, and decision analysis

Uncertainty in apparent performance and paired model differences was estimated using 1000 patient-level nonparametric bootstrap samples. Within each resample, component means and SDs were reestimated, candidate scores were reconstructed, and the corresponding Cox model and baseline hazard were refitted. Each bootstrap-fitted model was evaluated both in the bootstrap sample and in the original cohort using the transformations learned in the bootstrap sample. Mean training-minus-test optimism was subtracted from the original apparent estimate to obtain optimism-corrected performance. In parallel, stratified 10-fold cross-validation generated pooled OOF predictions. All component standardization, score scaling, model fitting, and baseline-hazard estimation were learned within each training fold and then applied unchanged to the held-out fold. Apparent estimates with percentile bootstrap 95% CIs, optimism-corrected estimates, and pooled OOF estimates were reported as complementary internal-validation perspectives. Calibration was evaluated at 3 and 5 years using the calibration intercept, calibration slope, integrated calibration index, observed-to-expected event ratio (O:E), and grouped plots comparing OOF predicted event risk with Kaplan–Meier observed event risk. Exploratory decision curve analysis used IPCW net benefit across threshold probabilities of 0.02 to 0.30. This range was selected for descriptive evaluation and did not represent validated clinical treatment thresholds.

### 2.7. Sensitivity analyses

All scoring systems were evaluated as candidate models in both score-only and pathology-adjusted models using the same bootstrap and 10-fold OOF procedures. Direct paired bootstrap differences were calculated for C-index, 3- and 5-year AUC, and 3- and 5-year Brier scores. Agreement between OOF 5-year event risks from the pathology + sIMIS and pathology + 4-component IMIS models was assessed continuously and after applying the 70th-percentile risk classification. To examine whether the incremental performance of sIMIS depended on an overly limited pathology comparator, an extended 8-variable clinicopathological reference model was evaluated. This model included age (continuous), tumor diameter ≥2 cm, non-endometrioid histology, abnormal p53 status, International Federation of Gynecology and Obstetrics (FIGO) stage III to IV, grade 3, LVSI, and myometrial invasion ≥50%, with and without the selected score.

### 2.8. Exploratory adjuvant treatment analyses

Treatment analyses were conducted after the prognostic analyses and were considered exploratory. Risk strata were defined using the 70th percentile of pooled OOF 5-year event risk from the pathology + sIMIS model. Any adjuvant therapy was defined as the logical OR of postoperative chemotherapy, radiotherapy, and combined chemoradiotherapy indicators. Within each OOF combined-model risk stratum, the propensity model included age, sIMIS, OOF 5-year risk, menopausal status, diabetes, hypertension, histology, p53 status, indicator terms for FIGO stages II, III, and IV, and grades 2 and 3, LVSI, myometrial invasion, Ki-67, and tumor diameter. An L2-penalized logistic model (*C* = 0.1) generated propensity scores. One-to-one matching without replacement used a 0.2 − SD caliper on the logit of the propensity score and mixed-integer cardinality optimization constrained every absolute standardized mean difference (SMD) to <0.10. Matched Kaplan–Meier curves were descriptive, and matched Cox models used pair-cluster robust standard errors. Formal treatment-by-risk interaction was evaluated in the full cohort. The adjusted model included age, menopausal status, diabetes, hypertension, LVSI, myometrial invasion, Ki-67, and tumor diameter. As a sensitivity analysis, overlap weighting reestimated an effectively unpenalized propensity model within each risk stratum using the same pretreatment covariates. Propensity-score overlap, effective sample size, and weighted SMDs were examined. Treatment initiation dates were unavailable, although DFS time began at surgery. The treatment analyses could therefore be affected by residual confounding, confounding by indication, treatment-modality heterogeneity, and immortal-time bias. They were not interpreted as estimates of causal treatment effects.

### 2.9. Statistical software

All tests were 2-sided. Statistical significance was defined as *P* < .05, but interpretation emphasized effect sizes, uncertainty, calibration, and consistency across validation methods rather than dichotomized *P* values. Analyses used Python 3.12 (Python Software Foundation), NumPy 2.3.5, pandas 2.2.3, SciPy 1.17.0, scikit-learn 1.8.0, matplotlib 3.10.8, and lifelines 0.30.0. The master random seed was 20,260,721, and the bootstrap stream seed was 20,260,722.

## 3. Results

### 3.1. Evaluation of candidate biomarkers and selection

All 4 literature-informed biomarkers were associated with DFS when modeled per 1 − SD risk-aligned increase. The HRs were 1.19 (95% CI, 1.03–1.38; *P* = .019) for SII, 1.36 (95% CI, 1.15–1.60; *P* < .001) for FIB, 1.63 (95% CI, 1.44–1.84; *P* < .001) for TG/HDL-C, and 1.66 (95% CI, 1.43–1.92; *P* < .001) for lower PNI (Table S2, Supplemental Digital Content 2). Among the individual markers, TG/HDL-C had the highest optimism-corrected and OOF C-indices (0.768 and 0.767, respectively). The 3 composite formulations showed broadly similar internal performance. Per 1 − SD risk-aligned increase, the HR was 1.69 (95% CI, 1.53–1.86) for sIMIS, 1.72 (95% CI, 1.54–1.92) for IMIS, and 1.68 (95% CI, 1.46–1.95) for the 2-component score (all *P* < .001). sIMIS had the largest likelihood-ratio chi-square statistic (68.46), compared with 62.56 for IMIS and 62.34 for the 2-component score. In score-only analyses, the optimism-corrected and OOF C-indices were 0.778 and 0.775 for sIMIS, 0.772 and 0.772 for IMIS, and 0.771 and 0.770 for the 2-component score. The corresponding optimism-corrected and OOF 3-year AUCs were 0.797 and 0.794, 0.792 and 0.791, and 0.790 and 0.788, respectively. At 5 years, the IMIS had a slightly higher AUC than sIMIS, indicating that no candidate dominated every measure (Table S2, Supplemental Digital Content 2). OOF calibration and exploratory decision curves likewise did not identify a consistent advantage for the 4-component formulation (Figs. S1 and S2, Supplemental Digital Content 3). sIMIS was carried forward because it had the largest likelihood-ratio statistic and numerically highest optimism-corrected and OOF C-indices while using 1 fewer component than the IMIS and retaining SII. The IMIS and 2-component score were retained as sensitivity formulations.

### 3.2. Cohort characteristics

The final cohort comprised 853 patients, of whom 94 (11.0%) experienced a DFS event. Reverse Kaplan–Meier median follow-up was 4.93 years. Mean age was 54.2 ± 8.3 years; 670 patients (78.5%) had FIGO stage I disease, 143 (16.8%) had grade 3 tumors, 91 (10.7%) had non-endometrioid histology, 84 (9.8%) had abnormal p53 status, and 255 (29.9%) had LVSI. Any adjuvant therapy was recorded for 405 patients (47.5%). The median raw composite scores were −0.334 (IQR, −1.218 to 0.764) for sIMIS and −0.436 (IQR, −1.538 to 0.984) for IMIS (Table 1).

**Table 1:** Baseline clinicopathological characteristics of the 853-patient cohort.

Characteristic	Level	Overall
Age, yr	Mean ± SD	54.2 ± 8.3
Menopausal status	Premenopausal	376 (44.1%)
Menopausal status	Postmenopausal	477 (55.9%)
Diabetes mellitus	No	781 (91.6%)
Diabetes mellitus	Yes	72 (8.4%)
Hypertension	No	626 (73.4%)
Hypertension	Yes	227 (26.6%)
Tumor diameter	<2 cm	203 (23.8%)
Tumor diameter	≥2 cm	650 (76.2%)
Histological type	Endometrioid	762 (89.3%)
Histological type	Non-endometrioid	91 (10.7%)
Ki-67 index	<60%	372 (43.6%)
Ki-67 index	≥60%	481 (56.4%)
p53 status	Wild-type	769 (90.2%)
p53 status	Abnormal	84 (9.8%)
FIGO stage	I	670 (78.5%)
FIGO stage	II	82 (9.6%)
FIGO stage	III	97 (11.4%)
FIGO stage	IV	4 (0.5%)
Tumor grade	Grade 1	544 (63.8%)
Tumor grade	Grade 2	166 (19.5%)
Tumor grade	Grade 3	143 (16.8%)
LVSI	Absent	598 (70.1%)
LVSI	Present	255 (29.9%)
Myometrial invasion	<50%	670 (78.5%)
Myometrial invasion	≥50%	183 (21.5%)
Adjuvant chemotherapy	No	523 (61.3%)
Adjuvant chemotherapy	Yes	330 (38.7%)
Adjuvant radiotherapy	No	661 (77.5%)
Adjuvant radiotherapy	Yes	192 (22.5%)
Combined chemoradiotherapy	No	736 (86.3%)
Combined chemoradiotherapy	Yes	117 (13.7%)
Any adjuvant therapy	No	448 (52.5%)
Any adjuvant therapy	Yes	405 (47.5%)
DFS event	Recurrence/progression or death	94 (11.0%)
Follow-up, years	Median (IQR), observed times	4.59 (3.83–5.54)
Follow-up, years	Reverse-KM median	4.93
sIMIS	Median (IQR)	−0.334 (−1.218 to 0.764)
IMIS	Median (IQR)	−0.436 (−1.538 to 0.984)

Note: Values are n (%) unless otherwise stated. The sIMIS and IMIS values shown are raw composite scores; HRs in Cox models are reported per 1 − SD increase in each score. Chemotherapy, radiotherapy, and combined chemoradiotherapy are overlapping modality indicators and are not mutually exclusive; any adjuvant therapy was defined from the logical OR of these fields. The IMIS distribution is shown only for sensitivity analysis. Follow-up was estimated by reverse Kaplan–Meier for the censoring distribution.

DFS = disease-free survival, FIGO = International Federation of Gynecology and Obstetrics, IMIS = immune-metabolic-inflammatory score, IQR = interquartile range, KM = Kaplan–Meier, LVSI = lymphovascular space invasion, SD = standard deviation, sIMIS = simplified immune-metabolic-inflammatory score.

### 3.3. Association of sIMIS with DFS

Each 1 − SD increase in sIMIS was associated with a higher DFS event hazard in the univariable model (HR, 1.69; 95% CI, 1.53–1.86; *P* < .001) and in the multivariable association model (HR, 1.65; 95% CI, 1.45–1.88; *P* < .001) (Table 2). Abnormal p53 status (HR, 3.60; 95% CI, 2.07–6.24), FIGO stage III to IV (HR, 2.79; 95% CI, 1.65–4.73), grade 3 disease (HR, 2.73; 95% CI, 1.54–4.85), and non-endometrioid histology (HR, 1.24; 95% CI, 1.06–1.45; *P* = .006) were also retained as adverse factors in the multivariable model. These 4 clinicopathological variables were therefore included in the pathology reference model. DFS decreased progressively across the quartile-based sIMIS strata. The low (*Q*1–*Q*2; n = 427), intermediate (*Q*3; n = 213), and high (*Q*4; n = 213) groups showed clearly separated Kaplan–Meier curves (log-rank *P* < .001; Fig. 1). The variable-level proportional hazards tests showed no evidence of nonproportionality for sIMIS in the multivariable association model (*P* = .220) or the primary combined model (*P* = .315). A potential violation was observed for myometrial invasion in its univariable model (*P* = .016), but not after multivariable adjustment (*P* = .071) (Table S3, Supplemental Digital Content 4).

**Table 2:** Univariable and multivariable Cox regression analyses for disease-free survival.

Variable	Contrast	Univariable HR (95% CI)	*P*	Multivariable HR (95% CI)	*P*
Age, yr	Per 1-yr increase	1.03 (1.01–1.06)	.011	1.06 (1.00–1.12)	.054
Postmenopausal status	Postmenopausal vs premenopausal	1.44 (0.94–2.19)	.091	–	–
Diabetes	Yes vs no	1.48 (0.79–2.78)	.221	–	–
Hypertension	Yes vs no	0.93 (0.58–1.47)	.751	–	–
Tumor diameter	≥2 cm vs <2 cm	1.78 (1.01–3.15)	.046	1.40 (0.74–2.65)	.297
Histological type	Non-endometrioid vs endometrioid	3.28 (2.06–5.21)	<.001	1.24 (1.06–1.45)	.006
Ki-67	≥60% vs <60%	1.15 (0.76–1.75)	.499	–	–
p53 status	Abnormal vs wild-type	5.52 (3.60–8.46)	<.001	3.60 (2.07–6.24)	<.001
FIGO stage	III–IV vs I–II	5.57 (3.68–8.42)	<.001	2.79 (1.65–4.73)	<.001
Tumor grade	Grade 3 vs grades 1–2	5.30 (3.54–7.94)	<.001	2.73 (1.54–4.85)	<.001
LVSI	Present vs absent	2.50 (1.66–3.74)	<.001	1.31 (0.80–2.15)	.279
Myometrial invasion	≥50% vs <50%	1.99 (1.30–3.04)	.002	0.73 (0.44–1.23)	.240
sIMIS	Per 1 − SD increase	1.69 (1.53–1.86)	<.001	1.65 (1.45–1.88)	<.001

The sIMIS effect is reported per 1 − SD increase. Variables with a univariable *P* value <.05 were entered into the exploratory multivariable association model.

CI = confidence interval, FIGO = International Federation of Gynecology and Obstetrics, HR = hazard ratio, LVSI = lymphovascular space invasion, SD = standard deviation, sIMIS = simplified immune-metabolic-inflammatory score.

### 3.4. Prognostic model performance, calibration, and decision analysis

**Figure 2.**
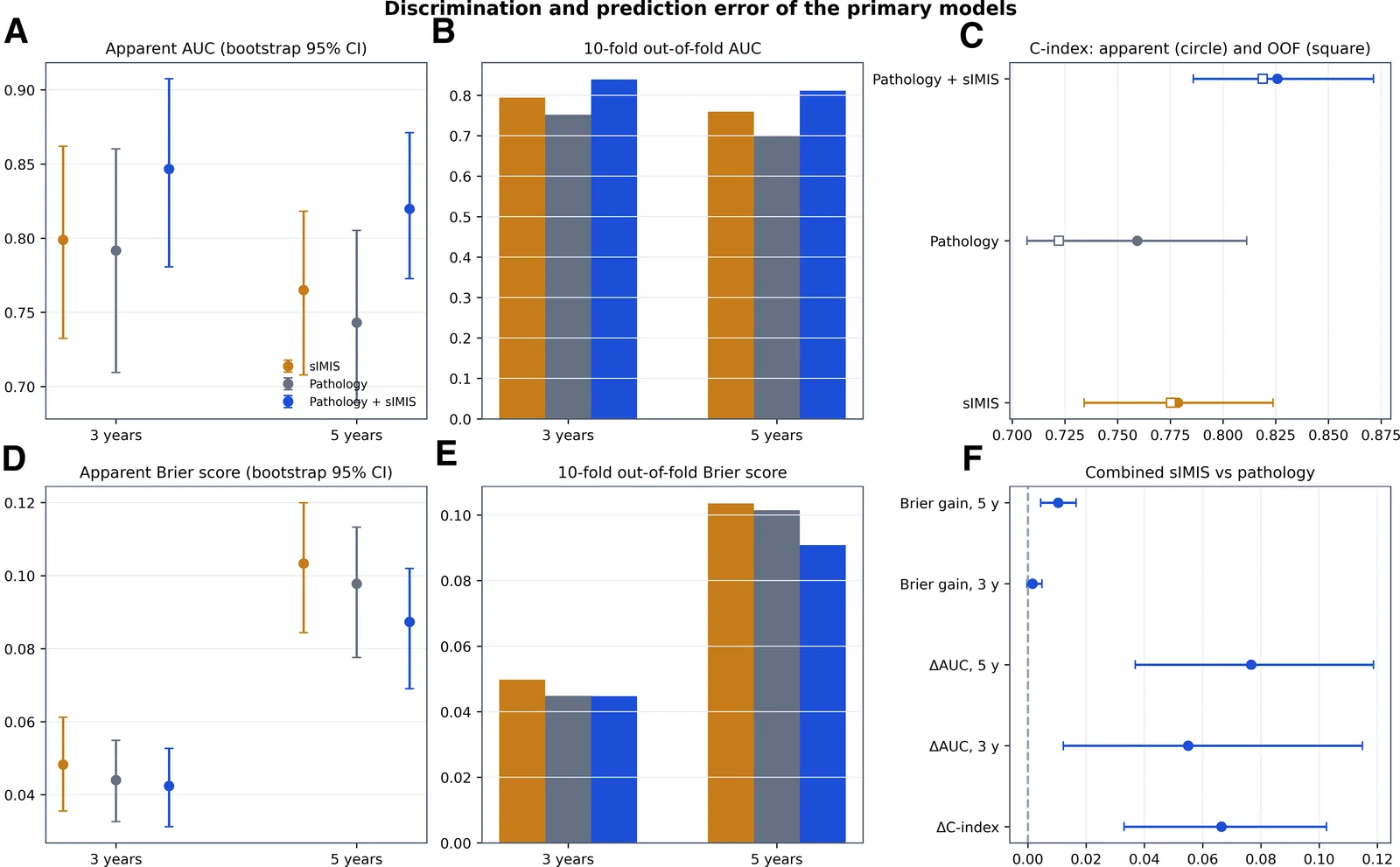
Discrimination and prediction error of the primary models. (A) Apparent 3- and 5-year AUCs with patient-level bootstrap 95% CIs. (B) Pooled 10-fold OOF 3- and 5-year AUCs. (C) Apparent C-indices (circles) with patient-level bootstrap 95% CIs and pooled 10-fold OOF C-indices (squares; point estimates only). (D) Apparent 3- and 5-year Brier scores with bootstrap 95% CIs. (E) Pooled 10-fold OOF Brier scores. (F) Apparent paired bootstrap differences for pathology + sIMIS compared with pathology alone; these are distinct from the separately reported optimism-corrected C-index difference. Positive Brier-score gains indicate lower prediction error for the combined model. AUCs = area under the time-dependent receiver operating characteristic curve, CIs = confidence intervals, DFS = disease-free survival, OOF = out-of-fold, sIMIS = simplified immune-metabolic-inflammatory score.

**Figure 3.**
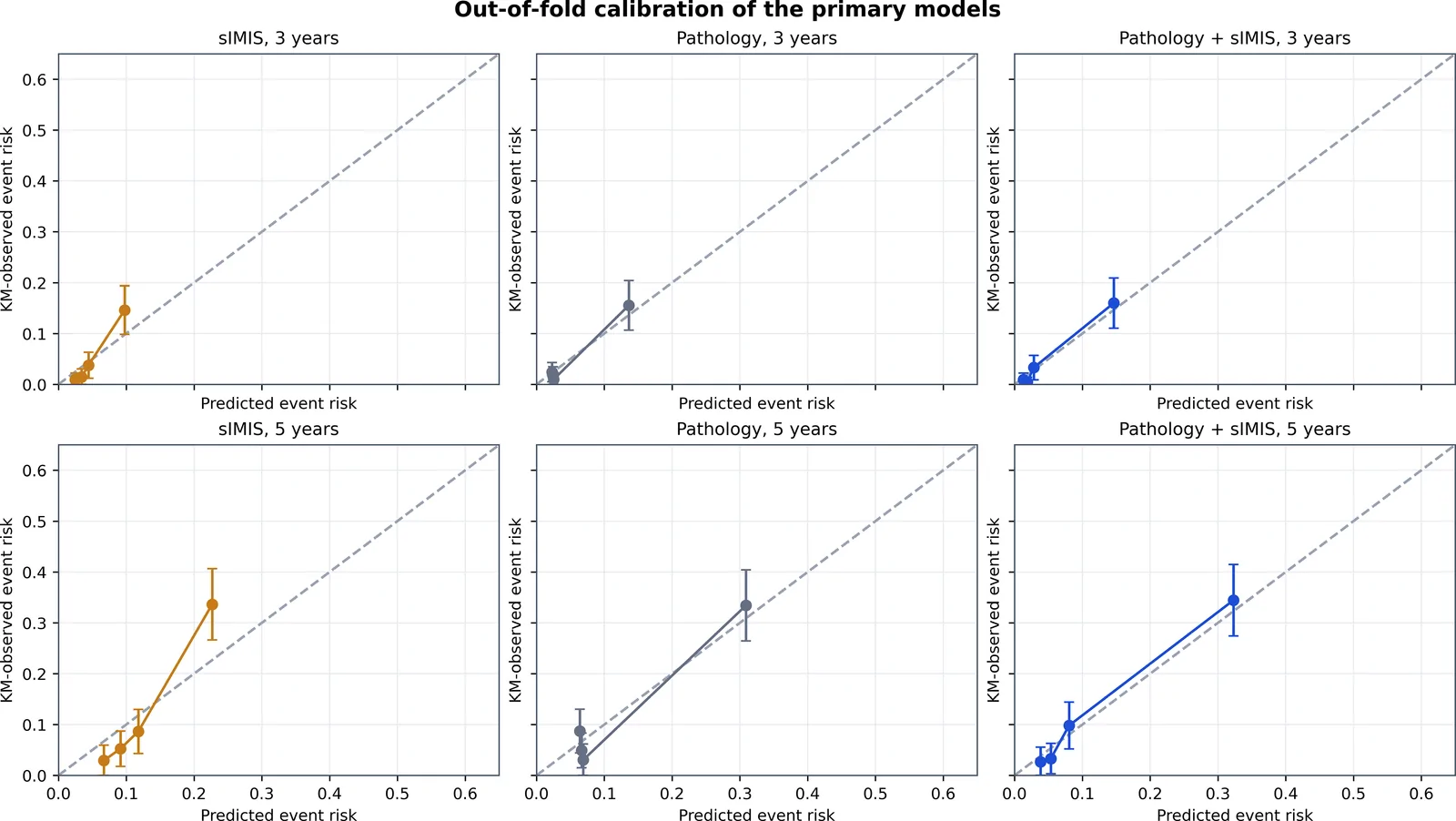
Out-of-fold calibration of the primary models. Pooled 10-fold OOF calibration of sIMIS-only, pathology-only, and pathology + sIMIS models at 3 years (top row) and 5 years (bottom row). Points represent grouped Kaplan–Meier observed event risk; error bars represent 95% CIs; the diagonal line indicates ideal calibration. CIs = confidence intervals, OOF = out-of-fold, sIMIS = simplified immune-metabolic-inflammatory score.

**Figure 4.**
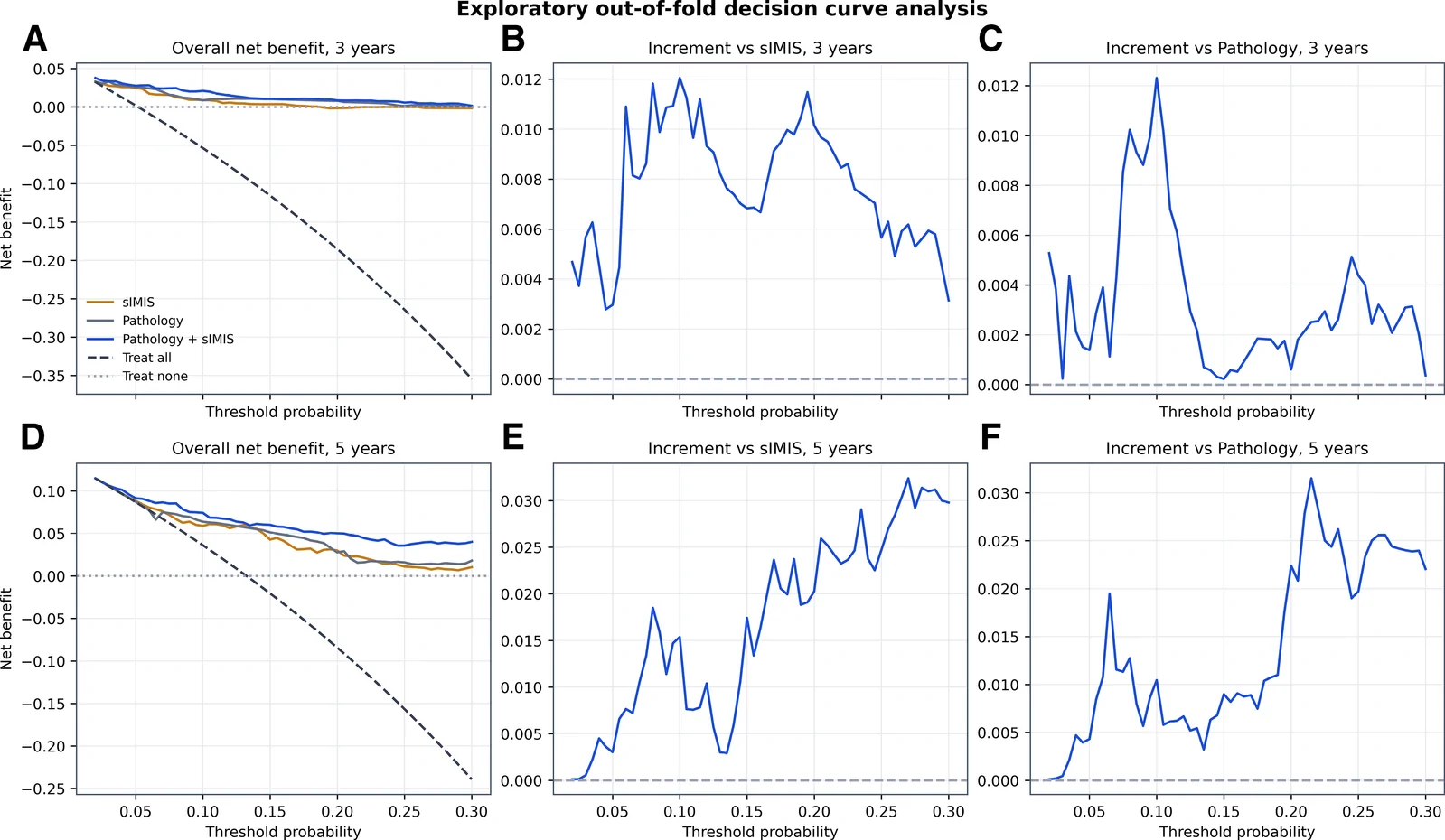
Exploratory out-of-fold decision curve analysis. Overall IPCW net benefit of the primary models at 3 years (A) and 5 years (D); incremental net benefit of pathology + sIMIS compared with sIMIS alone at 3 years (B) and 5 years (E); and incremental net benefit of pathology + sIMIS compared with pathology alone at 3 years (C) and 5 years (F). The assessed thresholds were exploratory and were not validated clinical action thresholds. IPCW = inverse probability of censoring weighting, sIMIS = simplified immune-metabolic-inflammatory score.

**Figure 5.**
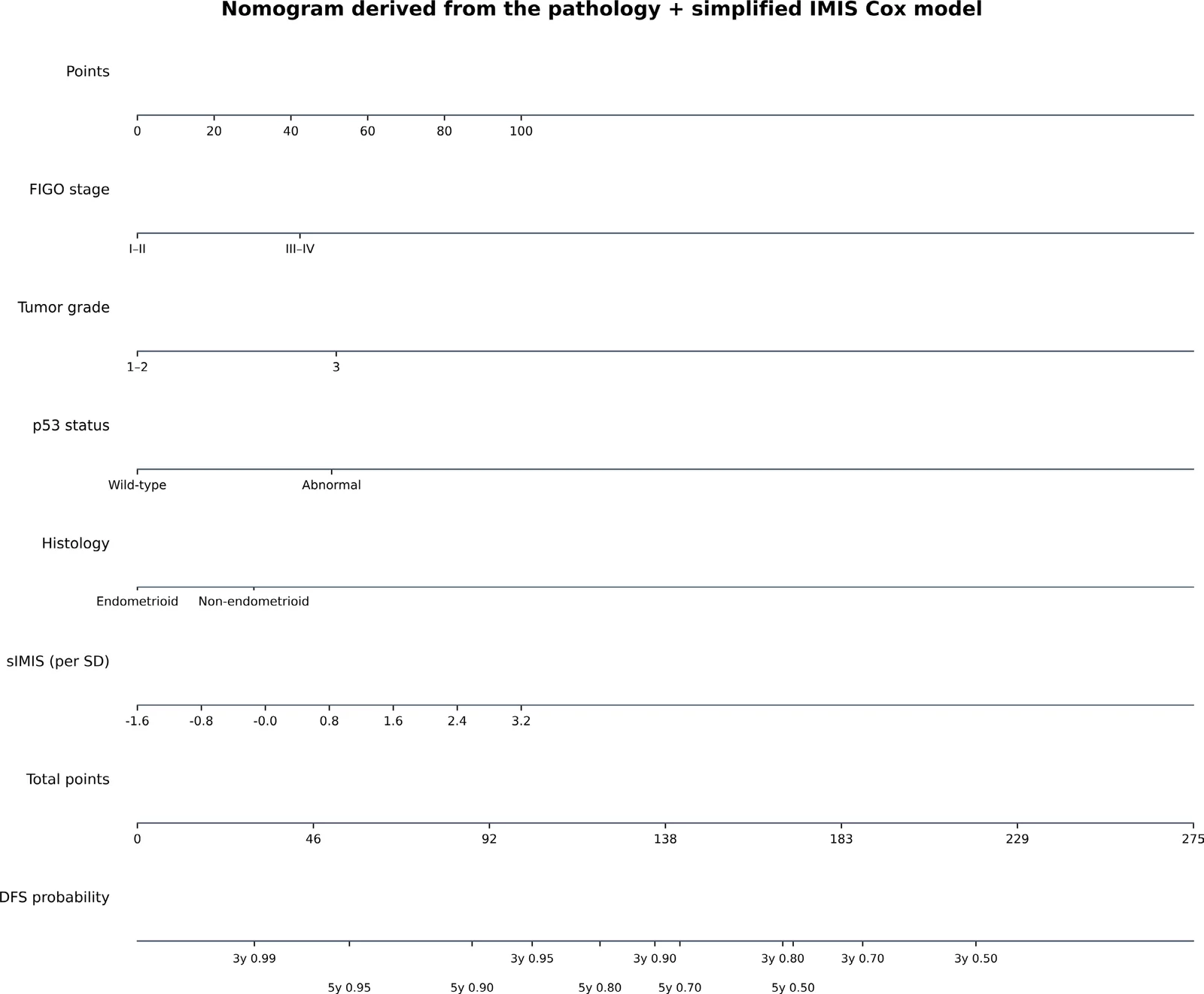
Nomogram derived from the pathology + sIMIS Cox model. Illustrative nomogram for 3- and 5-year DFS based on FIGO stage, tumor grade, p53 status, histological type, and sIMIS. The model was internally developed and has not undergone external validation. DFS = disease-free survival, FIGO = International Federation of Gynecology and Obstetrics, IMIS, immune-metabolic-inflammatory score, SD = standard deviation, sIMIS = simplified immune-metabolic-inflammatory score.

The pathology + sIMIS model had an apparent C-index of 0.826 (95% CI, 0.786–0.871), an optimism-corrected C-index of 0.820, and an OOF C-index of 0.819. Its optimism-corrected and OOF AUCs were 0.840 and 0.839 at 3 years, and 0.814 and 0.812 at 5 years. The corresponding optimism-corrected and OOF Brier scores were 0.044 and 0.045 at 3 years, and 0.091 and 0.091 at 5 years (Fig. 2 and Table S4A, Supplemental Digital Content 5). In apparent paired patient-level bootstrap comparisons with pathology alone, the C-index difference was 0.0664 (95% CI, 0.0330–0.1025), with an optimism-corrected difference of 0.0679. The 3- and 5-year AUC differences were 0.0550 (95% CI, 0.0122–0.1148) and 0.0767 (95% CI, 0.0368–0.1186), respectively. The 3-year Brier-score gain was small, and its CI included zero (0.0016; 95% CI, −0.0003 to 0.0048), whereas the 5-year gain was 0.0104 (95% CI, 0.0044–0.0165) (Table S4B, Supplemental Digital Content 6). Thus, the combined model showed supported gains in discrimination and 5-year prediction error, but not a clear improvement in every metric at both horizons. For the pathology + sIMIS model, apparent calibration intercepts at 3 and 5 years were − 0.009 (95% CI, −0.047 to 0.031) and −0.074 (95% CI, −0.138 to 0.004), with corresponding OOF estimates of −0.157 and −0.109. Apparent calibration slopes were 1.003 (95% CI, 0.867–1.145) and 0.971 (95% CI, 0.879–1.165), with OOF estimates of 0.818 and 0.882. Apparent integrated calibration indexes were 0.016 (95% CI, 0.011–0.028) and 0.024 (95% CI, 0.023–0.044), with OOF estimates of 0.017 and 0.024. Apparent O:E ratios were 1.021 (95% CI, 0.980–1.057) and 1.072 (95% CI, 1.034–1.114), with OOF estimates of 1.004 and 1.071, respectively (Fig. 3 and Table S7, Supplemental Digital Content 7). The OOF calibration slopes below 1 suggested some overfitting, while the 5-year O:E point estimate of 1.071 indicated modest underprediction at that horizon. Exploratory OOF decision curves showed generally positive incremental net benefit for the combined model across much of the assessed 0.02–0.30 threshold range (Fig. 4). Because no threshold represented a prespecified clinical action boundary, and uncertainty bands were not available, these curves were interpreted as exploratory rather than as proof of clinical utility. An illustrative nomogram based on the fitted pathology + sIMIS Cox model is shown in Figure 5; it represents the internally developed candidate model and is not a validated clinical calculator.

### 3.5. Sensitivity analyses

The IMIS did not show a consistent advantage over sIMIS. In score-only models, the optimism-corrected and OOF C-indices were 0.772 and 0.772 for the IMIS, compared with 0.778 and 0.775 for sIMIS. In pathology-adjusted models, the corresponding C-indices were 0.816 and 0.817 for pathology + IMIS and 0.820 and 0.819 for pathology + sIMIS. Paired differences in C-index and AUC between these formulations generally had CIs that included zero (Tables S4A and S4B, Supplemental Digital Content 6). OOF 5-year risk classification agreed for 809 of 853 patients (94.8%); 44 patients changed classification when the IMIS replaced sIMIS (Fig. S3, Supplemental Digital Content 8). The 2-component score also performed similarly but did not consistently exceed sIMIS. Its optimism-corrected and OOF C-indices were 0.771 and 0.770 alone and 0.815 and 0.815 when added to pathology, compared with 0.778 and 0.775 and 0.820 and 0.819, respectively, for sIMIS. Most paired C-index, AUC, and Brier-score differences had CIs that included zero (Tables S4A and S4B, Supplemental Digital Content 6). These comparisons support a parsimonious interpretation of sIMIS but do not establish it as the uniquely optimal formulation. When sIMIS was added to the extended 8-variable clinicopathological reference model, the OOF C-index increased from 0.746 to 0.816, the 3-year AUC from 0.765 to 0.829, and the 5-year AUC from 0.725 to 0.807. The 5-year Brier score decreased from 0.103 to 0.092, whereas the 3-year Brier score remained 0.046 (Table S10, Supplemental Digital Content 9). Thus, the observed increment was not confined to the more limited 4-variable pathology reference, although improvement remained metric- and horizon-dependent.

### 3.6. Exploratory adjuvant treatment analyses

**Figure 6.**
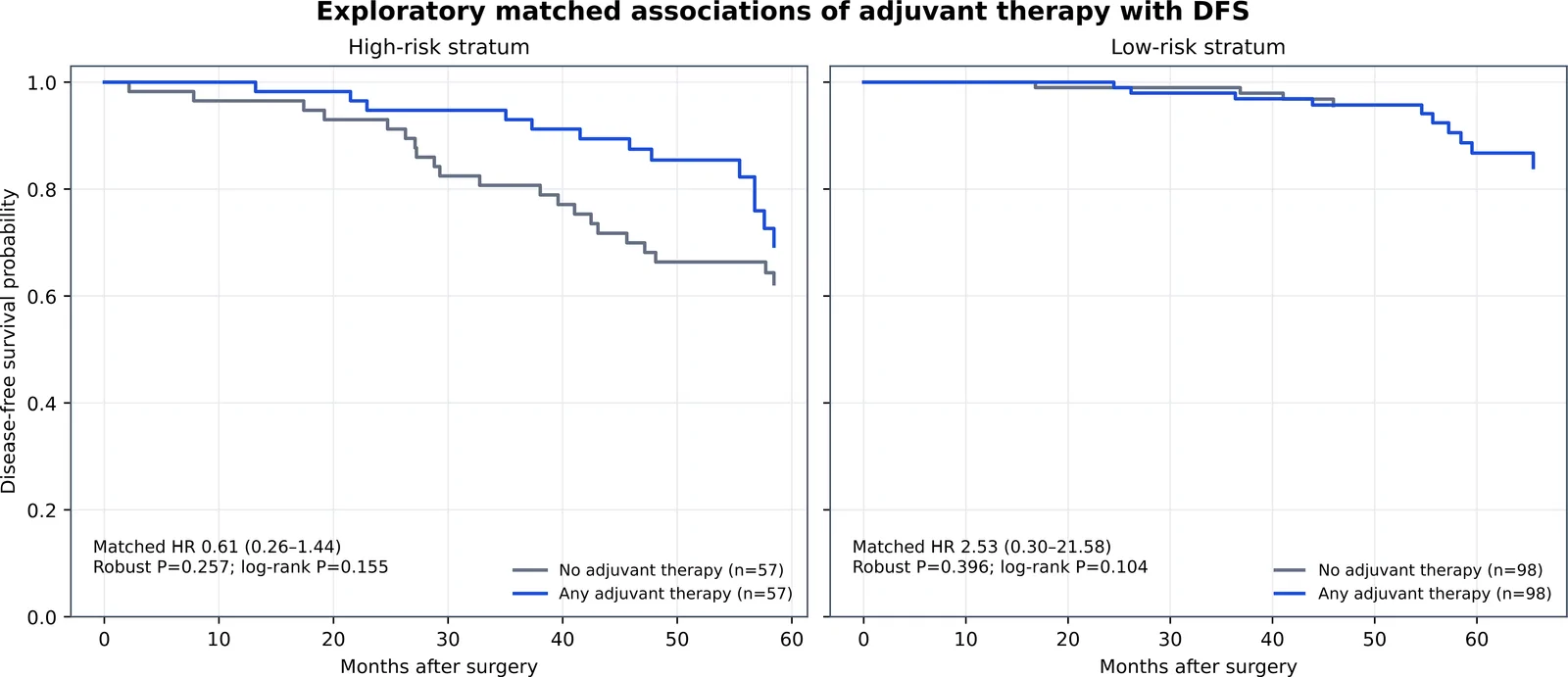
Exploratory matched associations of adjuvant therapy with disease-free survival. Kaplan–Meier comparisons by receipt of any adjuvant therapy in OOF pathology + sIMIS combined-model risk strata. Matching retained 57 pairs in the high-risk stratum (left panel) and 98 pairs in the low-risk stratum (right panel). Risk strata were defined using the 70th percentile of pooled OOF 5-year event risk and are distinct from the quartile-based sIMIS strata in Figure 1. DFS = disease-free survival, HR = hazard ratio, OOF = out-of-fold, sIMIS = simplified immune-metabolic-inflammatory score.

The 70th percentile of pooled OOF 5-year event risk from the pathology + sIMIS model was 0.095. This threshold classified 256 patients, with 73 DFS events, as high risk and 597 patients, with 21 events, as low risk. Matching retained 57 treated-untreated pairs in the high-risk stratum and 98 pairs in the low-risk stratum. All post-match absolute SMDs were <0.10, with a maximum of 0.098 in each stratum (Tables S5 and S6, Supplemental Digital Content 10; Fig. S4, Supplemental Digital Content 11). In the matched cohorts, the estimated HR comparing recorded adjuvant therapy with no recorded therapy was 0.61 (95% CI, 0.26–1.44; *P* = .257) in the high-risk stratum and 2.53 (95% CI, 0.30–21.58; *P* = .396) in the low-risk stratum (Fig. 6). Both estimates were imprecise, and neither provided evidence of an association within its stratum. Formal interaction tests did not support modification of the recorded treatment-DFS association by combined-model risk. In the covariate-adjusted full-cohort model, the therapy HRs were 0.61 (95% CI, 0.36–1.01) in the high-risk stratum and 1.40 (95% CI, 0.56–3.48) in the low-risk stratum, with *P* for interaction = .103. In the overlap-weighted analysis, the corresponding HRs were 0.67 (95% CI, 0.37–1.22) and 1.19 (95% CI, 0.11–13.01), with P for interaction = 0.663 (Table S8, Supplemental Digital Content 12). Overlap weighting achieved maximum absolute weighted SMDs of 0.001 in the high-risk stratum and 0.007 in the low-risk stratum, with effective sample sizes of 152.5 and 279.4, respectively (Tables S9A and S9B, Supplemental Digital Content 13). Nevertheless, propensity-score distributions showed limited common support, especially in the low-risk stratum (Fig. S5, Supplemental Digital Content 14). Taken together with the wide CIs, nonsignificant interactions, unavailable treatment dates, and potential confounding by indication, these findings did not provide robust evidence that combined-model risk modified the observed treatment-DFS association. The matched, adjusted, and overlap-weighted estimates are summarized in Figure S6, Supplemental Digital Content 15.

## 4. Discussion

In this single-center retrospective development cohort, we evaluated 4 literature-informed preoperative biomarkers and 3 risk-aligned equal-weight score formulations. All 4 biomarkers were individually associated with DFS. Among the candidate formulations, sIMIS was selected because it provided the most favorable balance of association strength, internally assessed discrimination, component count, and coverage of prespecified host-related domains; however, the alternative formulations performed broadly similarly, and no score was superior across all measures. In subsequent analyses, higher sIMIS was associated with a greater hazard of a DFS event after multivariable adjustment. Adding sIMIS to the 4-variable pathology reference model was associated with a higher C-index and time-dependent AUC and with a lower 5-year prediction error under internal resampling, although the 3-year Brier-score improvement was small and uncertain. Exploratory analyses did not provide robust evidence that combined-model risk modified the association between recorded adjuvant therapy and DFS.

The prognostic relevance of this signal is consistent with prior evidence across all 3 host-related domains represented in sIMIS. Elevated pretreatment SII has been associated with poorer outcomes in endometrial cancer cohorts,^[6,18]^ a finding reinforced by a recent disease-specific meta-analysis.^[19]^ Lower PNI has likewise been associated with adverse survival outcomes in pooled endometrial cancer data.^[20,21]^ For the metabolic domain, high triglyceride and low HDL-C levels were independently associated with poorer survival in a recent endometrial cancer cohort,^[22]^ while a composite containing TG/HDL-C and PNI also stratified survival in this disease.^[12]^ This convergence across inflammatory, metabolic, and immune-nutritional markers provides clinical support for the host-risk signal captured by sIMIS. The broadly similar results across the 3 prespecified formulations suggest that the observed signal was not driven solely by 1 particular score configuration, although external validation is needed to determine whether this consistency is reproducible.

This consistency is also biologically plausible. SII aggregates neutrophils, platelets, and lymphocytes and has repeatedly been associated with adverse endometrial cancer outcomes.^[18]^ More broadly, systemic inflammatory burden and tumor-associated myeloid immunometabolism can shape cancer progression,^[23,24]^ while relative lymphopenia may reflect reduced immune competence.^[7]^ TG/HDL-C summarizes an atherogenic dyslipidemic pattern linked to disturbed lipid metabolism, which can interact with immune function and the tumor microenvironment.^[25–28]^ PNI combines albumin and lymphocyte count and therefore reflects immune-nutritional reserve.^[4]^ High sIMIS can thus be interpreted as an integrated phenotype characterized by greater inflammatory and metabolic stress together with lower immune-nutritional reserve. These pathways provide plausible links between the systemic host environment and tumor progression, although the present data cannot establish mediation or determine whether the biomarker pattern is a cause or consequence of aggressive disease. Accordingly, sIMIS is best interpreted as an integrated prognostic phenotype rather than a mechanistic or therapeutic target.

On this basis, the principal clinical relevance of sIMIS may lie in its complementarity to tumor-centered risk assessment. Contemporary endometrial cancer stratification relies primarily on stage, histopathological features, and, increasingly, molecular classification.^[2,29]^ In contrast, sIMIS summarizes the patient’s systemic condition before surgery. The higher internally assessed performance observed after adding sIMIS to the primary and extended clinicopathological models suggests that sIMIS may capture complementary host-related information; this interpretation requires confirmation in external cohorts. Multi-institutional studies have likewise found that immune-inflammatory-nutritional indices contributed prognostic information alongside clinicopathological and ProMisE features.^[15,30]^ Rather than replacing established pathological or molecular factors, sIMIS should therefore be evaluated as an adjunct that may refine risk estimates within contemporary classification frameworks.

The exploratory treatment analyses addressed a related but conceptually distinct question. Whereas the prognostic analyses assessed whether sIMIS was associated with DFS, the treatment analyses examined whether risk defined by the combined model modified the association between recorded adjuvant therapy and DFS. In the high-risk stratum, estimates from matching, covariate adjustment, and overlap weighting were directionally similar (HRs, 0.61, 0.61, and 0.67, respectively), but their confidence intervals were wide. Formal interaction tests did not support effect modification in either the covariate-adjusted analysis (*P* for interaction = .103) or the overlap-weighted analysis (*P* for interaction = .663). The low-risk estimates were even less precise because few DFS events occurred. Thus, the directional pattern is hypothesis-generating but does not establish that sIMIS-defined risk predicts benefit from adjuvant therapy. Residual confounding by indication, treatment-modality heterogeneity, and limited propensity-score overlap constrain causal interpretation.^[31,32]^ Because treatment-initiation dates were unavailable while follow-up began at surgery, exposure assignment could not be aligned with time zero, leaving possible immortal-time bias.^[33]^ The main value of this exploratory analysis is therefore not to guide treatment decisions in the present cohort but to define a testable question for future studies with treatment-specific exposure data, molecular classification, and sufficient statistical power for interaction testing.

Beyond these treatment-specific concerns, the broader strengths and limitations of the study warrant consideration. Methodological strengths include the systematic evaluation of prespecified score formulations, the use of transparent risk-aligned equal weights, and complementary bootstrap and OOF procedures to quantify internally assessed performance. In addition, sIMIS is derived from routine preoperative laboratory measurements and does not require specialized assays or outcome-derived coefficients; if externally validated, it may offer a practical way to incorporate systemic host status into endometrial cancer risk assessment. These strengths should be weighed against several limitations. The retrospective, single-center design, modest number of DFS events, complete-case analysis, and use of the same cohort for candidate selection and internal evaluation may have introduced selection bias and optimistic performance estimates. Complete molecular classification was unavailable because only p53 status was recorded; polymerase epsilon exonuclease domain-mutated subtype mutation and mismatch-repair status were not captured. The extent of LVSI was unavailable, and a single preoperative measurement may not capture changes in host status over time. In addition, OOF calibration slopes below 1 and deviations in the calibration intercept and O:E ratio indicated residual internal miscalibration. The present results therefore support the prognostic association and internally assessed performance of sIMIS but not its transportability, absolute-risk calibration in other settings, or incremental value beyond molecular classification. Accordingly, the fixed sIMIS formula should next be externally evaluated in geographically diverse and molecularly characterized cohorts. Such studies should assess reproducibility, calibration and any need for recalibration, and incremental value within contemporary molecular risk groups. If externally confirmed, sIMIS could serve as a pragmatic host-based complement to established pathological and molecular approaches.

## 5. Conclusion

In this development cohort, higher sIMIS was associated with poorer DFS after multivariable adjustment. Adding sIMIS to the primary and extended clinicopathological models yielded higher internally assessed discrimination, although the gains were metric- and horizon-dependent and require external confirmation. These findings identify sIMIS as a candidate, routinely available host-based prognostic marker rather than an established clinical or treatment-selection tool. External validation in molecularly characterized cohorts is needed to assess transportability, absolute-risk calibration, and incremental value within contemporary risk systems. The exploratory treatment analyses did not provide evidence of a treatment-by-risk interaction and should remain hypothesis-generating.

## Author contributions

**Conceptualization:** Shuhuan Li.

**Data curation:** Shuhuan Li, Yuer Sun, Jiayu Chen, Jing Yan, Miaomiao Nie, Mei Wu.

**Formal analysis:** Shuhuan Li.

**Funding acquisition:** Shuhuan Li, Juan Li, Zhenzhen Wu.

**Methodology:** Shuhuan Li.

**Project administration:** Juan Li, Zhenzhen Wu.

**Resources:** Juan Li.

**Software:** Shuhuan Li.

**Supervision:** Yawen Shao, Zhenzhen Wu.

**Visualization:** Shuhuan Li.

**Writing – original draft:** Shuhuan Li.

**Writing – review & editing:** Yawen Shao, Zhenzhen Wu.




































