# Supplementary material for: Incremental prognostic value of a preoperative immune-metabolic-inflammatory score for disease-free survival in endometrial cancer: A retrospective cohort study
Source: Medicine (Baltimore). 2026 Aug 21;105(34):e50354. doi: 10.1097/MD.0000000000050354 (PMC13502846; doi:10.1097/MD.0000000000050354)
Supplement: Supplementary file 1 [file medi-105-e50354-s001.docx]

**Table S1. Standardization parameters and formulas for sIMIS and IMIS**

| **Component** | **Definition** | **Mean** | **SD** |
| --- | --- | --- | --- |
| SII | PLT × NEU / LYM | 596.333434 | 435.164043 |
| TG/HDL-C | TG / HDL-C | 1.286248 | 0.951495 |
| PNI | ALB + 5 × LYM | 53.801758 | 5.305216 |
| FIB | Fibrinogen | 2.760539 | 0.690794 |
| Two-component score | z(TG/HDL-C) − z(PNI) | 0.000 | 1.274808 |
| sIMIS raw score | z(SII)+z(TG/HDL-C)−z(PNI) | 0.000 | 1.931523 |
| IMIS raw score | z(SII)+z(FIB)+z(TG/HDL-C)−z(PNI) | 0.000 | 2.368014 |

Note: z(x) = (x − mean)/SD. Laboratory units were PLT, NEU, and LYM in ×10⁹/L; ALB and FIB in g/L; and TG and HDL-C in mmol/L. Means and SDs were re-estimated within each bootstrap sample and cross-validation training fold; the full-cohort values shown here define the final candidate formulas. The table reports raw composite scores; for Cox analyses, each score was rescaled to its own SD.
