# Supplementary material for: Incremental prognostic value of a preoperative immune-metabolic-inflammatory score for disease-free survival in endometrial cancer: A retrospective cohort study
Source: Medicine (Baltimore). 2026 Aug 21;105(34):e50354. doi: 10.1097/MD.0000000000050354 (PMC13502846; doi:10.1097/MD.0000000000050354)
Supplement: Supplementary file 2 [file medi-105-e50354-s002.docx]

**Table S2. Associations and predictive performance of individual components and score formulations**

| **Marker/score** | **HR per SD (95% CI)** | **P** | **LR χ²; P** | **C-index** | **3-y AUC** | **5-y AUC** | **3-y Brier** | **5-y Brier** |
| --- | --- | --- | --- | --- | --- | --- | --- | --- |
| SII | 1.19 (1.03–1.38) | 0.019 | 4.31; 0.038 | 0.540 (0.480–0.610); corrected 0.533; OOF 0.537 | corrected 0.565; OOF 0.562 | corrected 0.505; OOF 0.507 | corrected 0.049; OOF 0.049 | corrected 0.115; OOF 0.114 |
| Fibrinogen | 1.36 (1.15–1.60) | <0.001 | 11.30; <0.001 | 0.615 (0.554–0.672); corrected 0.616; OOF 0.614 | corrected 0.616; OOF 0.609 | corrected 0.623; OOF 0.620 | corrected 0.049; OOF 0.049 | corrected 0.114; OOF 0.112 |
| TG/HDL-C | 1.63 (1.44–1.84) | <0.001 | 60.36; <0.001 | 0.770 (0.714–0.812); corrected 0.768; OOF 0.767 | corrected 0.775; OOF 0.773 | corrected 0.756; OOF 0.755 | corrected 0.049; OOF 0.049 | corrected 0.105; OOF 0.104 |
| Lower PNI | 1.66 (1.43–1.92) | <0.001 | 33.07; <0.001 | 0.658 (0.598–0.718); corrected 0.658; OOF 0.656 | corrected 0.660; OOF 0.656 | corrected 0.650; OOF 0.648 | corrected 0.047; OOF 0.047 | corrected 0.111; OOF 0.110 |
| Two-component score [z(TG/HDL-C) − z(PNI)] | 1.68 (1.46–1.95) | <0.001 | 62.34; <0.001 | 0.772 (0.722–0.813); corrected 0.771; OOF 0.770 | corrected 0.790; OOF 0.788 | corrected 0.763; OOF 0.761 | corrected 0.049; OOF 0.049 | corrected 0.105; OOF 0.104 |
| sIMIS | 1.69 (1.53–1.86) | <0.001 | 68.46; <0.001 | 0.779 (0.734–0.824); corrected 0.778; OOF 0.775 | corrected 0.797; OOF 0.794 | corrected 0.765; OOF 0.760 | corrected 0.049; OOF 0.050 | corrected 0.105; OOF 0.104 |
| IMIS | 1.72 (1.54–1.92) | <0.001 | 62.56; <0.001 | 0.773 (0.726–0.816); corrected 0.772; OOF 0.772 | corrected 0.792; OOF 0.791 | corrected 0.772; OOF 0.770 | corrected 0.049; OOF 0.050 | corrected 0.104; OOF 0.103 |

Note: The C-index cell gives the apparent estimate with bootstrap 95% CI, followed by the optimism-corrected and 10-fold OOF estimates. The two-component score is z(TG/HDL-C) − z(PNI), not a subtraction in the original measurement units. Lower PNI is aligned to the risk direction.
