# Supplementary material for: Incremental prognostic value of a preoperative immune-metabolic-inflammatory score for disease-free survival in endometrial cancer: A retrospective cohort study
Source: Medicine (Baltimore). 2026 Aug 21;105(34):e50354. doi: 10.1097/MD.0000000000050354 (PMC13502846; doi:10.1097/MD.0000000000050354)
Supplement: Supplementary file 3 [file medi-105-e50354-s004.docx]

**Table S3. Scaled Schoenfeld residual tests for proportional hazards**

| **Model** | **Variable** | **χ²** | **df** | **P** |
| --- | --- | --- | --- | --- |
| Univariable | Age, years | 0.617 | 1 | 0.432 |
| Univariable | Postmenopausal | 0.000 | 1 | 0.997 |
| Univariable | Diabetes | 1.792 | 1 | 0.181 |
| Univariable | Hypertension | 1.562 | 1 | 0.211 |
| Univariable | Tumor diameter ≥2 cm | 0.002 | 1 | 0.964 |
| Univariable | Non-endometrioid histology | 0.490 | 1 | 0.484 |
| Univariable | Ki-67 ≥60% | 0.122 | 1 | 0.726 |
| Univariable | p53 abnormal | 0.012 | 1 | 0.912 |
| Univariable | FIGO III–IV | 1.046 | 1 | 0.306 |
| Univariable | Grade 3 | 1.929 | 1 | 0.165 |
| Univariable | LVSI present | 3.129 | 1 | 0.077 |
| Univariable | Myometrial invasion ≥50% | 5.844 | 1 | 0.016 |
| Univariable | sIMIS per SD | 1.002 | 1 | 0.317 |
| Table 2 multivariable association model | Age, years | 2.437 | 1 | 0.119 |
| Table 2 multivariable association model | Tumor diameter ≥2 cm | 0.752 | 1 | 0.386 |
| Table 2 multivariable association model | Non-endometrioid histology | 0.073 | 1 | 0.786 |
| Table 2 multivariable association model | p53 abnormal | 0.898 | 1 | 0.343 |
| Table 2 multivariable association model | FIGO III–IV | 0.281 | 1 | 0.596 |
| Table 2 multivariable association model | Grade 3 | 0.746 | 1 | 0.388 |
| Table 2 multivariable association model | LVSI present | 0.414 | 1 | 0.520 |
| Table 2 multivariable association model | Myometrial invasion ≥50% | 3.253 | 1 | 0.071 |
| Table 2 multivariable association model | sIMIS per SD | 1.505 | 1 | 0.220 |
| Pathology-only prediction model | FIGO III–IV | 0.173 | 1 | 0.677 |
| Pathology-only prediction model | Grade 3 | 1.312 | 1 | 0.252 |
| Pathology-only prediction model | p53 abnormal | 0.507 | 1 | 0.477 |
| Pathology-only prediction model | Non-endometrioid | 0.046 | 1 | 0.830 |
| Primary combined model | FIGO III–IV | 0.308 | 1 | 0.579 |
| Primary combined model | Grade 3 | 1.495 | 1 | 0.221 |
| Primary combined model | p53 abnormal | 0.668 | 1 | 0.414 |
| Primary combined model | Non-endometrioid | 0.073 | 1 | 0.787 |
| Primary combined model | sIMIS per SD | 1.009 | 1 | 0.315 |
| IMIS sensitivity model | FIGO III–IV | 0.590 | 1 | 0.443 |
| IMIS sensitivity model | Grade 3 | 1.994 | 1 | 0.158 |
| IMIS sensitivity model | p53 abnormal | 0.402 | 1 | 0.526 |
| IMIS sensitivity model | Non-endometrioid | 0.302 | 1 | 0.583 |
| IMIS sensitivity model | IMIS per SD | 1.059 | 1 | 0.303 |

Note: Variable-level tests use the validated lifelines proportional_hazard_test implementation with the rank time transform when available. Global rows are explicitly labeled as approximate sums of variable-level statistics. A P value <0.05 indicates potential non-proportionality. df, degrees of freedom.
