# Supplementary material for: Incremental prognostic value of a preoperative immune-metabolic-inflammatory score for disease-free survival in endometrial cancer: A retrospective cohort study
Source: Medicine (Baltimore). 2026 Aug 21;105(34):e50354. doi: 10.1097/MD.0000000000050354 (PMC13502846; doi:10.1097/MD.0000000000050354)
Supplement: Supplementary file 4 [file medi-105-e50354-s005.docx]

**Table S4A. Primary and score-formulation sensitivity model performance**

| **Model** | **C-index** | **3-y AUC** | **5-y AUC** | **3-y Brier** | **5-y Brier** |
| --- | --- | --- | --- | --- | --- |
| sIMIS-only | 0.779 (0.734–0.824); corrected 0.778; OOF 0.775 | 0.799 (0.733–0.862); corrected 0.797; OOF 0.794 | 0.765 (0.708–0.818); corrected 0.765; OOF 0.760 | 0.048 (0.036–0.061); corrected 0.049; OOF 0.050 | 0.103 (0.084–0.120); corrected 0.105; OOF 0.104 |
| Pathology-only | 0.759 (0.707–0.811); corrected 0.752; OOF 0.722 | 0.792 (0.709–0.860); corrected 0.784; OOF 0.752 | 0.743 (0.689–0.805); corrected 0.736; OOF 0.699 | 0.044 (0.033–0.055); corrected 0.045; OOF 0.045 | 0.098 (0.078–0.113); corrected 0.101; OOF 0.101 |
| Pathology + sIMIS | 0.826 (0.786–0.871); corrected 0.820; OOF 0.819 | 0.847 (0.781–0.907); corrected 0.840; OOF 0.839 | 0.820 (0.773–0.871); corrected 0.814; OOF 0.812 | 0.042 (0.031–0.053); corrected 0.044; OOF 0.045 | 0.087 (0.069–0.102); corrected 0.091; OOF 0.091 |
| IMIS-only | 0.773 (0.726–0.816); corrected 0.772; OOF 0.772 | 0.793 (0.733–0.853); corrected 0.792; OOF 0.791 | 0.772 (0.713–0.822); corrected 0.772; OOF 0.770 | 0.049 (0.036–0.062); corrected 0.049; OOF 0.050 | 0.103 (0.084–0.120); corrected 0.104; OOF 0.103 |
| Pathology + IMIS | 0.822 (0.783–0.865); corrected 0.816; OOF 0.817 | 0.844 (0.779–0.902); corrected 0.837; OOF 0.837 | 0.818 (0.773–0.869); corrected 0.812; OOF 0.811 | 0.043 (0.032–0.054); corrected 0.045; OOF 0.045 | 0.090 (0.071–0.105); corrected 0.093; OOF 0.093 |
| Two-component score | 0.772 (0.722–0.813); corrected 0.771; OOF 0.770 | 0.791 (0.730–0.856); corrected 0.790; OOF 0.788 | 0.765 (0.710–0.808); corrected 0.763; OOF 0.761 | 0.049 (0.033–0.058); corrected 0.049; OOF 0.049 | 0.103 (0.084–0.116); corrected 0.105; OOF 0.104 |
| Pathology + two-component score | 0.820 (0.786–0.854); corrected 0.815; OOF 0.815 | 0.840 (0.765–0.896); corrected 0.834; OOF 0.833 | 0.815 (0.770–0.865); corrected 0.811; OOF 0.808 | 0.043 (0.033–0.054); corrected 0.044; OOF 0.044 | 0.092 (0.075–0.109); corrected 0.096; OOF 0.095 |

Note: Each cell reports apparent performance (bootstrap 95% CI), optimism-corrected performance, and pooled 10-fold OOF performance. IMIS and two-component models are sensitivity analyses.
