# Supplementary material for: Incremental prognostic value of a preoperative immune-metabolic-inflammatory score for disease-free survival in endometrial cancer: A retrospective cohort study
Source: Medicine (Baltimore). 2026 Aug 21;105(34):e50354. doi: 10.1097/MD.0000000000050354 (PMC13502846; doi:10.1097/MD.0000000000050354)
Supplement: Supplementary file 5 [file medi-105-e50354-s006.docx]

**Table S4B. Paired bootstrap differences among primary and sensitivity models**

| **Comparison** | **Metric** | **Apparent paired difference (95% CI)** | **Optimism-corrected difference** | **Paired replicates** | **Direction** |
| --- | --- | --- | --- | --- | --- |
| Pathology + sIMIS vs Pathology-only | C-index | 0.0664 (0.0330 to 0.1025) | 0.0679 | 1,000 | First model minus reference; positive favors first model |
| Pathology + sIMIS vs Pathology-only | 3-y AUC | 0.0550 (0.0122 to 0.1148) | 0.0559 | 1,000 | First model minus reference; positive favors first model |
| Pathology + sIMIS vs Pathology-only | 5-y AUC | 0.0767 (0.0368 to 0.1186) | 0.0785 | 1,000 | First model minus reference; positive favors first model |
| Pathology + sIMIS vs Pathology-only | 3-y Brier | 0.0016 (-0.0003 to 0.0048) | 0.0012 | 1,000 | Reference minus first model; positive favors first model |
| Pathology + sIMIS vs Pathology-only | 5-y Brier | 0.0104 (0.0044 to 0.0165) | 0.0105 | 1,000 | Reference minus first model; positive favors first model |
| Pathology + sIMIS vs sIMIS-only | C-index | 0.0471 (0.0183 to 0.0845) | 0.0422 | 1,000 | First model minus reference; positive favors first model |
| Pathology + sIMIS vs sIMIS-only | 3-y AUC | 0.0478 (0.0025 to 0.0965) | 0.0428 | 1,000 | First model minus reference; positive favors first model |
| Pathology + sIMIS vs sIMIS-only | 5-y AUC | 0.0547 (0.0141 to 0.1062) | 0.0494 | 1,000 | First model minus reference; positive favors first model |
| Pathology + sIMIS vs sIMIS-only | 3-y Brier | 0.0058 (0.0021 to 0.0123) | 0.0048 | 1,000 | Reference minus first model; positive favors first model |
| Pathology + sIMIS vs sIMIS-only | 5-y Brier | 0.0160 (0.0077 to 0.0286) | 0.0139 | 1,000 | Reference minus first model; positive favors first model |
| IMIS-only vs sIMIS-only | C-index | -0.0059 (-0.0283 to 0.0167) | -0.0054 | 1,000 | First model minus reference; positive favors first model |
| IMIS-only vs sIMIS-only | 3-y AUC | -0.0059 (-0.0347 to 0.0241) | -0.0051 | 1,000 | First model minus reference; positive favors first model |
| IMIS-only vs sIMIS-only | 5-y AUC | 0.0066 (-0.0201 to 0.0350) | 0.0069 | 1,000 | First model minus reference; positive favors first model |
| IMIS-only vs sIMIS-only | 3-y Brier | -0.0003 (-0.0017 to 0.0003) | -0.0003 | 1,000 | Reference minus first model; positive favors first model |
| IMIS-only vs sIMIS-only | 5-y Brier | 0.0004 (-0.0024 to 0.0039) | 0.0005 | 1,000 | Reference minus first model; positive favors first model |

| **Comparison** | **Metric** | **Apparent paired difference (95% CI)** | **Optimism-corrected difference** | **Paired replicates** | **Direction** |
| --- | --- | --- | --- | --- | --- |
| Pathology + IMIS vs Pathology + sIMIS | C-index | -0.0033 (-0.0168 to 0.0094) | -0.0039 | 1,000 | First model minus reference; positive favors first model |
| Pathology + IMIS vs Pathology + sIMIS | 3-y AUC | -0.0025 (-0.0217 to 0.0165) | -0.0028 | 1,000 | First model minus reference; positive favors first model |
| Pathology + IMIS vs Pathology + sIMIS | 5-y AUC | -0.0014 (-0.0167 to 0.0150) | -0.0020 | 1,000 | First model minus reference; positive favors first model |
| Pathology + IMIS vs Pathology + sIMIS | 3-y Brier | -0.0010 (-0.0024 to -0.0002) | -0.0008 | 1,000 | Reference minus first model; positive favors first model |
| Pathology + IMIS vs Pathology + sIMIS | 5-y Brier | -0.0024 (-0.0051 to 0.0003) | -0.0023 | 1,000 | Reference minus first model; positive favors first model |
| sIMIS vs two-component score | C-index | 0.0070 (-0.0197 to 0.0333) | 0.0070 | 1,000 | First model minus reference; positive favors first model |
| sIMIS vs two-component score | 3-y AUC | 0.0081 (-0.0252 to 0.0412) | 0.0071 | 1,000 | First model minus reference; positive favors first model |
| sIMIS vs two-component score | 5-y AUC | 0.0000 (-0.0287 to 0.0286) | 0.0020 | 1,000 | First model minus reference; positive favors first model |
| sIMIS vs two-component score | 3-y Brier | 0.0010 (-0.0024 to 0.0044) | 0.0000 | 1,000 | Reference minus first model; positive favors first model |
| sIMIS vs two-component score | 5-y Brier | 0.0001 (-0.0070 to 0.0076) | 0.0000 | 1,000 | Reference minus first model; positive favors first model |
| Pathology + sIMIS vs Pathology + two-component score | C-index | 0.0062 (-0.0115 to 0.0238) | 0.0050 | 1,000 | First model minus reference; positive favors first model |
| Pathology + sIMIS vs Pathology + two-component score | 3-y AUC | 0.0071 (-0.0115 to 0.0254) | 0.0061 | 1,000 | First model minus reference; positive favors first model |
| Pathology + sIMIS vs Pathology + two-component score | 5-y AUC | 0.0050 (-0.0151 to 0.0258) | 0.0032 | 1,000 | First model minus reference; positive favors first model |
| Pathology + sIMIS vs Pathology + two-component score | 3-y Brier | 0.0012 (-0.0014 to 0.0041) | 0.0000 | 1,000 | Reference minus first model; positive favors first model |
| Pathology + sIMIS vs Pathology + two-component score | 5-y Brier | 0.0054 (0.0000 to 0.0119) | 0.0051 | 1,000 | Reference minus first model; positive favors first model |

Note: Differences were calculated within each of 1,000 patient-level bootstrap resamples. Finite-resolution sign-test P values are omitted; inference should emphasize paired 95% CIs. For Brier score, positive values denote lower error for the first model, as stated in the Direction column.
