# Supplementary material for: Incremental prognostic value of a preoperative immune-metabolic-inflammatory score for disease-free survival in endometrial cancer: A retrospective cohort study
Source: Medicine (Baltimore). 2026 Aug 21;105(34):e50354. doi: 10.1097/MD.0000000000050354 (PMC13502846; doi:10.1097/MD.0000000000050354)
Supplement: Supplementary file 6 [file medi-105-e50354-s007.docx]

**Table S7. Calibration of the primary models**

| **Model** | **Horizon** | **Intercept** | **Slope** | **ICI** | **O:E** |
| --- | --- | --- | --- | --- | --- |
| sIMIS-only | 3 years | -0.003 (-0.011 to 0.008); OOF -0.406 | 0.955 (0.840 to 1.148); OOF 0.605 | 0.030 (0.018 to 0.046); OOF 0.029 | 1.045 (1.007 to 1.070); OOF 1.033 |
| sIMIS-only | 5 years | 0.000 (-0.082 to 0.038); OOF 0.006 | 1.369 (0.879 to 1.747); OOF 1.344 | 0.051 (0.031 to 0.074); OOF 0.049 | 1.048 (1.024 to 1.079); OOF 1.054 |
| Pathology-only | 3 years | -0.008 (-0.050 to 0.023); OOF -0.022 | 1.030 (0.857 to 1.202); OOF 0.968 | 0.014 (0.006 to 0.028); OOF 0.015 | 1.000 (0.964 to 1.031); OOF 0.996 |
| Pathology-only | 5 years | -0.084 (-0.143 to -0.016); OOF -0.111 | 0.896 (0.805 to 1.029); OOF 0.818 | 0.017 (0.011 to 0.036); OOF 0.020 | 1.045 (1.014 to 1.079); OOF 1.044 |
| Pathology + sIMIS | 3 years | -0.009 (-0.047 to 0.031); OOF -0.157 | 1.003 (0.867 to 1.145); OOF 0.818 | 0.016 (0.011 to 0.028); OOF 0.017 | 1.021 (0.980 to 1.057); OOF 1.004 |
| Pathology + sIMIS | 5 years | -0.074 (-0.138 to 0.004); OOF -0.109 | 0.971 (0.879 to 1.165); OOF 0.882 | 0.024 (0.023 to 0.044); OOF 0.024 | 1.072 (1.034 to 1.114); OOF 1.071 |

Note: Each cell reports the apparent estimate with bootstrap 95% CI and the pooled 10-fold OOF estimate. CI, confidence interval; ICI, integrated calibration index; O:E, observed-to-expected event ratio; OOF, out-of-fold.
