# Supplementary material for: Incremental prognostic value of a preoperative immune-metabolic-inflammatory score for disease-free survival in endometrial cancer: A retrospective cohort study
Source: Medicine (Baltimore). 2026 Aug 21;105(34):e50354. doi: 10.1097/MD.0000000000050354 (PMC13502846; doi:10.1097/MD.0000000000050354)
Supplement: Supplementary file 7 [file medi-105-e50354-s009.docx]

**Table S10. Sensitivity analysis using an extended clinicopathological reference model**

| **Model** | **Parameters** | **Incremental LR χ²; P** | **AIC** | **OOF C-index** | **OOF 3-y AUC** | **OOF 5-y AUC** | **OOF 3-y Brier** | **OOF 5-y Brier** | **Covariates** |
| --- | --- | --- | --- | --- | --- | --- | --- | --- | --- |
| Extended reference | 8 | Reference | 1122.37 | 0.746 | 0.765 | 0.725 | 0.046 | 0.103 | Age, tumor diameter, histology, p53, FIGO stage, grade, LVSI, myometrial invasion |
| Extended reference + sIMIS | 9 | 44.30; <0.001 | 1080.07 | 0.816 | 0.829 | 0.807 | 0.046 | 0.092 | Age, tumor diameter, histology, p53, FIGO stage, grade, LVSI, myometrial invasion, sIMIS |

Note: The extended reference model included age (continuous), tumor diameter ≥2 cm, non-endometrioid histology, abnormal p53 status, FIGO stage III–IV, grade 3, LVSI present, and myometrial invasion ≥50%. The extended reference + sIMIS model added sIMIS per 1-SD increase. Standardization of sIMIS was learned within each cross-validation training fold. This analysis evaluates whether the sIMIS increment was an artifact of an overly limited four-variable pathology comparator. AIC, Akaike information criterion.
