# Supplementary material for: Incremental prognostic value of a preoperative immune-metabolic-inflammatory score for disease-free survival in endometrial cancer: A retrospective cohort study
Source: Medicine (Baltimore). 2026 Aug 21;105(34):e50354. doi: 10.1097/MD.0000000000050354 (PMC13502846; doi:10.1097/MD.0000000000050354)
Supplement: Supplementary file 8 [file medi-105-e50354-s010.docx]

**Table S5. Covariate balance in the high-risk stratum defined by OOF 5-year event risk from the pathology+sIMIS combined model**

| **Covariate** | **Before: no therapy** | **Before: therapy** | **After: no therapy** | **After: therapy** | Signed SMD before | Signed SMD after |
| --- | --- | --- | --- | --- | --- | --- |
| Age, years | 52.551 ± 8.897 | 55.843 ± 8.925 | 54.421 ± 8.529 | 53.632 ± 8.827 | 0.369 | -0.089 |
| sIMIS (SD units) | 1.292 ± 1.229 | 0.554 ± 1.223 | 0.938 ± 0.923 | 0.887 ± 1.277 | -0.602 | -0.042 |
| OOF-predicted 5-year event risk | 0.260 ± 0.218 | 0.299 ± 0.213 | 0.247 ± 0.205 | 0.226 ± 0.141 | 0.180 | -0.098 |
| Postmenopausal | 40/78 (51.3%) | 111/178 (62.4%) | 33/57 (57.9%) | 31/57 (54.4%) | 0.224 | -0.071 |
| Diabetes | 6/78 (7.7%) | 17/178 (9.6%) | 4/57 (7.0%) | 3/57 (5.3%) | 0.066 | -0.062 |
| Hypertension | 24/78 (30.8%) | 49/178 (27.5%) | 17/57 (29.8%) | 19/57 (33.3%) | -0.071 | 0.077 |
| Non-endometrioid histology | 8/78 (10.3%) | 64/178 (36.0%) | 7/57 (12.3%) | 9/57 (15.8%) | 0.638 | 0.087 |
| p53 abnormal | 22/78 (28.2%) | 60/178 (33.7%) | 17/57 (29.8%) | 17/57 (29.8%) | 0.119 | 0.000 |
| FIGO II | 4/78 (5.1%) | 13/178 (7.3%) | 4/57 (7.0%) | 5/57 (8.8%) | 0.090 | 0.072 |
| FIGO III | 10/78 (12.8%) | 84/178 (47.2%) | 10/57 (17.5%) | 11/57 (19.3%) | 0.806 | 0.041 |
| FIGO IV | 1/78 (1.3%) | 3/178 (1.7%) | 1/57 (1.8%) | 1/57 (1.8%) | 0.033 | 0.000 |
| Grade 2 | 11/78 (14.1%) | 33/178 (18.5%) | 10/57 (17.5%) | 12/57 (21.1%) | 0.120 | 0.095 |
| Grade 3 | 26/78 (33.3%) | 106/178 (59.6%) | 22/57 (38.6%) | 21/57 (36.8%) | 0.542 | -0.036 |
| LVSI present | 17/78 (21.8%) | 95/178 (53.4%) | 17/57 (29.8%) | 16/57 (28.1%) | 0.687 | -0.038 |
| Myometrial invasion ≥50% | 14/78 (17.9%) | 73/178 (41.0%) | 14/57 (24.6%) | 12/57 (21.1%) | 0.521 | -0.079 |
| Ki-67 ≥60% | 44/78 (56.4%) | 129/178 (72.5%) | 34/57 (59.6%) | 36/57 (63.2%) | 0.339 | 0.074 |
| Tumor diameter ≥2 cm | 59/78 (75.6%) | 149/178 (83.7%) | 46/57 (80.7%) | 48/57 (84.2%) | 0.200 | 0.087 |

Note: Risk strata were defined using the 70th percentile of pooled OOF 5-year event risk from the pathology+sIMIS combined model. Continuous variables are mean ± SD; binary variables are n/N (%). The SMD columns report signed values; balance was defined as |SMD| < 0.10.
