# Supplementary material for: Incremental prognostic value of a preoperative immune-metabolic-inflammatory score for disease-free survival in endometrial cancer: A retrospective cohort study
Source: Medicine (Baltimore). 2026 Aug 21;105(34):e50354. doi: 10.1097/MD.0000000000050354 (PMC13502846; doi:10.1097/MD.0000000000050354)
Supplement: Supplementary file 9 [file medi-105-e50354-s012.docx]

**Table S8. Treatment-by-risk interaction analyses**

| **Analysis** | **Estimate** | **HR/ratio (95% CI)** | **P** | **P interaction** |
| --- | --- | --- | --- | --- |
| Unadjusted full-cohort Cox | Therapy HR in low-risk group | 2.03 (0.85–4.82) | 0.109 | — |
| Unadjusted full-cohort Cox | Therapy HR in high-risk group | 0.80 (0.49–1.29) | 0.356 | — |
| Unadjusted full-cohort Cox | Ratio of therapy HRs (high vs low) | 0.39 (0.15–1.06) | 0.064 | 0.064 |
| Covariate-adjusted full-cohort Cox | Therapy HR in low-risk group | 1.40 (0.56–3.48) | 0.470 | — |
| Covariate-adjusted full-cohort Cox | Therapy HR in high-risk group | 0.61 (0.36–1.01) | 0.057 | — |
| Covariate-adjusted full-cohort Cox | Ratio of therapy HRs (high vs low) | 0.43 (0.16–1.18) | 0.103 | 0.103 |
| Overlap-weighted Cox (primary interaction sensitivity analysis) | Therapy HR in low-risk group | 1.19 (0.11–13.01) | 0.886 | — |
| Overlap-weighted Cox (primary interaction sensitivity analysis) | Therapy HR in high-risk group | 0.67 (0.37–1.22) | 0.191 | — |
| Overlap-weighted Cox (primary interaction sensitivity analysis) | Ratio of therapy HRs (high vs low) | 0.56 (0.04–7.47) | 0.663 | 0.663 |

Note: Risk strata were defined by the 70th percentile (0.095) of pooled 10-fold OOF 5-year event risk from the pathology+sIMIS model (high risk: n = 256, 73 DFS events; low risk: n = 597, 21 DFS events). HRs compare any recorded adjuvant therapy with no recorded therapy. The ratio of therapy HRs is high-risk/low-risk, and P for interaction is from the therapy-by-risk-stratum term. These analyses should not be interpreted causally because treatment initiation dates were unavailable.
