# Supplementary material for: Incremental prognostic value of a preoperative immune-metabolic-inflammatory score for disease-free survival in endometrial cancer: A retrospective cohort study
Source: Medicine (Baltimore). 2026 Aug 21;105(34):e50354. doi: 10.1097/MD.0000000000050354 (PMC13502846; doi:10.1097/MD.0000000000050354)
Supplement: Supplementary file 10 [file medi-105-e50354-s013.docx]

**Table S9B. Covariate balance after overlap weighting**

| **Risk stratum** | **Covariate** | Signed SMD unweighted | Signed SMD weighted |
| --- | --- | --- | --- |
| low | Age, years | 0.329 | 0.004 |
| low | sIMIS | 0.126 | 0.000 |
| low | OOF-predicted 5-year event risk | 0.177 | -0.007 |
| low | Postmenopausal | 0.245 | 0.001 |
| low | Diabetes | 0.010 | 0.000 |
| low | Hypertension | 0.152 | 0.002 |
| low | Non-endometrioid histology | 0.393 | 0.003 |
| low | p53 abnormal | 0.133 | 0.000 |
| low | FIGO II | 0.487 | 0.002 |
| low | FIGO III | 0.163 | 0.000 |
| low | FIGO IV | 0.000 | 0.000 |
| low | Grade 2 | 0.588 | 0.000 |
| low | Grade 3 | 0.276 | 0.003 |
| low | LVSI present | 1.410 | 0.000 |
| low | Myometrial invasion ≥50% | 0.637 | 0.000 |
| low | Ki-67 ≥60% | 0.198 | 0.000 |
| low | Tumor diameter ≥2 cm | 0.575 | -0.001 |

| **Risk stratum** | **Covariate** | Signed SMD unweighted | Signed SMD weighted |
| --- | --- | --- | --- |
| high | Age, years | 0.369 | 0.001 |
| high | sIMIS | -0.602 | 0.000 |
| high | OOF-predicted 5-year event risk | 0.180 | 0.000 |
| high | Postmenopausal | 0.224 | 0.001 |
| high | Diabetes | 0.066 | 0.001 |
| high | Hypertension | -0.071 | 0.000 |
| high | Non-endometrioid histology | 0.638 | 0.000 |
| high | p53 abnormal | 0.119 | 0.000 |
| high | FIGO II | 0.090 | -0.001 |
| high | FIGO III | 0.806 | 0.001 |
| high | FIGO IV | 0.033 | -0.001 |
| high | Grade 2 | 0.120 | 0.001 |
| high | Grade 3 | 0.542 | 0.000 |
| high | LVSI present | 0.687 | 0.000 |
| high | Myometrial invasion ≥50% | 0.521 | 0.000 |
| high | Ki-67 ≥60% | 0.339 | 0.000 |
| high | Tumor diameter ≥2 cm | 0.200 | 0.000 |

Note: The SMD columns report signed values; the prespecified balance target was |SMD| < 0.10. Any residual imbalance should be considered when interpreting treatment associations.
