# Supplementary material for: Incremental prognostic value of a preoperative immune-metabolic-inflammatory score for disease-free survival in endometrial cancer: A retrospective cohort study
Source: Medicine (Baltimore). 2026 Aug 21;105(34):e50354. doi: 10.1097/MD.0000000000050354 (PMC13502846; doi:10.1097/MD.0000000000050354)
Supplement: Supplementary file 11 [file medi-105-e50354-s017.docx]

**Table S6. Covariate balance in the low-risk stratum defined by OOF 5-year event risk from the pathology+sIMIS combined model**

| **Covariate** | **Before: no therapy** | **Before: therapy** | **After: no therapy** | **After: therapy** | Signed SMD before | Signed SMD after |
| --- | --- | --- | --- | --- | --- | --- |
| Age, years | 52.968 ± 8.052 | 55.555 ± 7.666 | 55.969 ± 8.716 | 55.204 ± 8.140 | 0.329 | -0.097 |
| sIMIS (SD units) | -0.363 ± 0.617 | -0.287 ± 0.589 | -0.293 ± 0.623 | -0.296 ± 0.554 | 0.126 | -0.006 |
| OOF-predicted 5-year event risk | 0.053 ± 0.015 | 0.056 ± 0.017 | 0.055 ± 0.015 | 0.053 ± 0.015 | 0.177 | -0.098 |
| Postmenopausal | 185/370 (50.0%) | 141/227 (62.1%) | 60/98 (61.2%) | 56/98 (57.1%) | 0.245 | -0.083 |
| Diabetes | 30/370 (8.1%) | 19/227 (8.4%) | 6/98 (6.1%) | 4/98 (4.1%) | 0.010 | -0.074 |
| Hypertension | 86/370 (23.2%) | 68/227 (30.0%) | 31/98 (31.6%) | 27/98 (27.6%) | 0.152 | -0.092 |
| Non-endometrioid histology | 1/370 (0.3%) | 18/227 (7.9%) | 1/98 (1.0%) | 2/98 (2.0%) | 0.393 | 0.052 |
| p53 abnormal | 0/370 (0.0%) | 2/227 (0.9%) | 0/98 (0.0%) | 0/98 (0.0%) | 0.133 | 0.000 |
| FIGO II | 18/370 (4.9%) | 47/227 (20.7%) | 18/98 (18.4%) | 21/98 (21.4%) | 0.487 | 0.094 |
| FIGO III | 0/370 (0.0%) | 3/227 (1.3%) | 0/98 (0.0%) | 0/98 (0.0%) | 0.163 | 0.000 |
| FIGO IV | 0/370 (0.0%) | 0/227 (0.0%) | 0/98 (0.0%) | 0/98 (0.0%) | 0.000 | 0.000 |
| Grade 2 | 42/370 (11.4%) | 80/227 (35.2%) | 38/98 (38.8%) | 35/98 (35.7%) | 0.588 | -0.075 |
| Grade 3 | 1/370 (0.3%) | 10/227 (4.4%) | 1/98 (1.0%) | 0/98 (0.0%) | 0.276 | -0.068 |
| LVSI present | 14/370 (3.8%) | 129/227 (56.8%) | 14/98 (14.3%) | 17/98 (17.3%) | 1.410 | 0.081 |
| Myometrial invasion ≥50% | 26/370 (7.0%) | 70/227 (30.8%) | 26/98 (26.5%) | 29/98 (29.6%) | 0.637 | 0.082 |
| Ki-67 ≥60% | 177/370 (47.8%) | 131/227 (57.7%) | 60/98 (61.2%) | 56/98 (57.1%) | 0.198 | -0.082 |
| Tumor diameter ≥2 cm | 239/370 (64.6%) | 200/227 (88.1%) | 87/98 (88.8%) | 84/98 (85.7%) | 0.575 | -0.075 |

Note: Risk strata were defined using the 70th percentile of pooled OOF 5-year event risk from the pathology+sIMIS combined model. Continuous variables are mean ± SD; binary variables are n/N (%). The SMD columns report signed values; balance was defined as |SMD| < 0.10.
