# Supplementary material for: Incremental prognostic value of a preoperative immune-metabolic-inflammatory score for disease-free survival in endometrial cancer: A retrospective cohort study
Source: Medicine (Baltimore). 2026 Aug 21;105(34):e50354. doi: 10.1097/MD.0000000000050354 (PMC13502846; doi:10.1097/MD.0000000000050354)
Supplement: Supplementary file 12 [file medi-105-e50354-s018.docx]

**Table S9A. Overlap-weight diagnostics by OOF risk stratum**

| **Risk stratum** | **OOF 5-y risk cutoff** | **Patients** | **DFS events** | **Effective sample size** | **Maximum absolute weighted SMD** | **Minimum propensity score** | **Maximum propensity score** |
| --- | --- | --- | --- | --- | --- | --- | --- |
| low | 0.095 | 597 | 21 | 279.391 | 0.007 | 0.026 | 1.000 |
| high | 0.095 | 256 | 73 | 152.543 | 0.001 | 0.033 | 0.992 |

Note: Risk strata were defined by the 70th percentile (0.095) of pooled OOF 5-year event risk from the pathology+sIMIS model (high risk: n = 256, 73 DFS events; low risk: n = 597, 21 DFS events). Propensity scores were estimated within each risk stratum; overlap weights target patients with treatment equipoise.
